# Tatton-Brown-Rahman Syndrome Due to a Novel DNMT3A Variant Presenting With Autism, Attention-Deficit/Hyperactivity Disorder (ADHD), and Regression: A Saudi Case Report

**DOI:** 10.7759/cureus.96388

**Published:** 2025-11-08

**Authors:** Abdulkarim O Alanazi, Enas M Aljohani, Sari Enani

**Affiliations:** 1 Psychiatry Department, Prince Sultan Military Medical City, Riyadh, SAU

**Keywords:** asd, autism spectrum disorder, dnmt3a, intellectual disability, overgrowth syndrome, tatton–brown–rahman syndrome, tbrs

## Abstract

Tatton-Brown-Rahman syndrome (TBRS) is a rare overgrowth/intellectual disability disorder caused by DNMT3A variants. It is characterized by tall stature, macrocephaly, and intellectual disability, with an expanding neuropsychiatric spectrum that includes autism spectrum disorder (ASD) and behavioral disturbances. We describe a young adult Saudi male with overgrowth, macrocephaly, coarse facial features, and global developmental delay. Cognitive testing confirmed mild intellectual disability with relative verbal strengths. He was diagnosed with ASD and attention-deficit/hyperactivity disorder (ADHD) and treated with methylphenidate in childhood. In early adulthood, the patient exhibited regression characterized by speech loss, decline in self-care, incontinence, and psychotic-like symptoms, alongside gastrointestinal disease. Genetic testing revealed a novel heterozygous DNMT3A missense variant (c.923G>T, p.Gly308Val) in the PWWP domain, consistent with TBRS. This case illustrates the broadened phenotype of TBRS, including ASD, ADHD, and regression, paralleling prior reports of cognitive unevenness and behavioral vulnerability. Additional findings of eczema, elevated IgE, and anemia suggest possible underrecognized systemic involvement. This case emphasizes the importance of multidisciplinary evaluation and lifelong neuropsychiatric follow-up in TBRS. As the first reported case from Saudi Arabia to our knowledge, it broadens the clinical and geographic spectrum of the disorder and highlights the association between DNMT3A variants, intellectual disability, and ASD.

## Introduction

Overgrowth syndromes constitute a heterogeneous, rare group of genetic disorders in which weight, height, and/or head circumference typically exceed the 97th centile above two standard deviations for age and sex, with substantial phenotypic overlap that complicates clinical recognition [1]. Many overgrowth entities arise from the disruption of epigenetic regulators of growth and differentiation, including NSD1, EZH2, and DNMT3A, among others [2]. Within this spectrum, Tatton-Brown-Rahman syndrome (TBRS) was delineated in 2014 in individuals with overgrowth harboring heterozygous variants in DNMT3A [2]. Clinically, TBRS overlaps with Sotos, Weaver, and Malan syndromes; however, it is genetically distinct from these conditions. TBRS results from pathogenic DNMT3A variants, whereas Sotos and Weaver are associated with NSD1 and EZH2, respectively [1,2].

TBRS constitutes a rare syndrome characterized by overgrowth and intellectual disability (OGID), predominantly resulting from de novo heterozygous mutations in the DNMT3A gene [3]. The principal clinical characteristics comprise increased height, enlarged head circumference, cognitive impairment, and a distinctive facial configuration characterized by broad horizontal eyebrows and pronounced upper central incisors [4]. The neuropsychiatric spectrum of TBRS is becoming more well acknowledged; in the most significant clinical cohort, behavioral and mental health issues were prevalent, with around 54% of patients having hypotonia and 22% having afebrile seizures [3]. Additional reports describe behavioral disturbances and psychotic symptoms in some individuals, underscoring the importance of incorporating routine neuropsychiatric assessments into standard pediatric and genetic evaluations [1]. In addition to neurodevelopmental and behavioral manifestations, systemic findings in TBRS may include joint hypermobility, congenital heart defects, neuroimaging abnormalities, and genitourinary anomalies such as cryptorchidism and vesicoureteral reflux [1-5]. These features, variably reported across cohorts, may account for urinary symptoms and further emphasize the multisystemic nature of DNMT3A-related disease.

Despite the emerging association between TBRS and autism spectrum disorder (ASD), detailed child-focused descriptions and genotype-phenotype correlations for neurobehavioral outcomes remain limited, and further systematically characterized cases are needed [1]. Moreover, recent case literature continues to emphasize that the phenotype and its delineation are still under study, underscoring ongoing knowledge gaps in natural history and neurodevelopmental profiling [2]. Reports of TBRS have been documented across Europe, Latin America, and Asia, demonstrating its recognition in multiple regions worldwide [1,2].

This case aims to contribute to the limited literature on TBRS by presenting a clinically and genetically supported case from Saudi Arabia. By highlighting the coexistence of TBRS with ASD, intellectual disability, attention-deficit/hyperactivity disorder (ADHD), and possible regression in early adulthood, we seek to expand the understanding of the neuropsychiatric spectrum of this rare overgrowth syndrome and emphasize the importance of multidisciplinary evaluation and management.

## Case presentation

The patient is a young adult Saudi male, born at term to consanguineous parents following an uncomplicated pregnancy and delivery. Early developmental delays were evident, with late acquisition of both motor and speech milestones. He achieved independent walking later than expected and produced his first words only around the age of four to five years. The patient also had delayed toilet training, and throughout childhood, he demonstrated limited academic ability, never acquiring functional literacy or numeracy.

Formal cognitive assessment revealed a verbal IQ of 67, a performance IQ of 46, and a full-scale IQ of 53, indicating overall intellectual functioning within the mild intellectual disability range. The test was administered under standard clinical procedures, and no copyrighted materials or test items were reproduced; use complied with the publisher’s terms. These findings place him within the category of mild intellectual disability, with a notable discrepancy between verbal and performance abilities, suggesting relative preservation of verbal comprehension compared to non-verbal and practical reasoning.

A diagnosis of ASD was made clinically based on developmental history and direct examination, without formal psychometric testing. He consistently demonstrated impaired social reciprocity, minimal eye contact, and a markedly reduced use of gestures and facial expressions in social interactions. Verbal communication was limited, with delayed language development and restricted conversational ability, while nonverbal communication remained poorly integrated. He exhibited stereotyped motor movements and repetitive use of objects, with restricted interests often focused on narrow and repetitive themes, accompanied by sensory sensitivities to sound and touch. Deficits in imaginative and pretend play were observed, and functional skills remained limited despite age. Taken together, these features encompassed the domains of social communication deficits and restricted, repetitive patterns of behavior, interests, and activities, all of which fulfilled the Diagnostic and Statistical Manual of Mental Disorders, Fifth Edition (DSM-5) diagnostic criteria for ASD [6].

The diagnosis of ADHD was established based on clinical evaluation and standardized rating scales, which documented persistent symptoms of hyperactivity, impulsivity, distractibility, and difficulty sustaining attention across multiple settings since early childhood, significantly impairing academic and social functioning. For ADHD, he had been treated during his school years with methylphenidate up to 36 mg daily, which provided reasonable control of hyperactivity until its discontinuation at the age of 18.

The family resides in a rural area, which has posed significant challenges in accessing specialized medical and psychiatric interventions throughout the patient’s developmental years and continues to limit timely follow-up and comprehensive multidisciplinary care.

From a psychiatric perspective, his course has been marked by pervasive developmental impairment with the later emergence of concerning behavioral changes in early adulthood. Caregivers described a gradual but marked regression in speech, behavior, and self-care, with verbal output reduced to mostly single-word utterances, accompanied by repetitive stereotyped movements. He also developed persistent non-purposeful roaming around the house and frequent episodes of self-talk, often coupled with incongruent affect such as inappropriate laughter or crying. These features raised the differential of psychotic-like symptoms versus manifestations of autistic behavior, including stereotyped self-dialogue or solitary imaginative play. Functional decline was further highlighted by the loss of previously acquired skills, including writing, and the onset of both nocturnal and diurnal urinary incontinence, along with severe sleep disturbance characterized by only a few hours of rest at night and continuous wandering during the day. In addition, caregivers noted episodes of sudden irritability with unprovoked aggression, including hitting family members, which significantly increased caregiver burden. In parallel, he experienced significant unintentional weight loss, dropping from approximately 100 kg to 69 kg, which was later correlated with gastrointestinal pathology, including *Helicobacter pylori* gastritis and active proctitis, identified on endoscopic and histopathological evaluation. Collectively, these findings raised the possibility of regression syndrome occurring in the context of ASD.

Psychopharmacological interventions were trialed at different stages. Risperidone was introduced at a dose of 0.5 mL at bedtime, resulting in improved sleep and partial behavioral stabilization, with no adverse effects reported. Previous trials of olanzapine had been discontinued due to significant weight gain, while aripiprazole was attempted prior to olanzapine but led to intolerable akathisia. No history of seizures was elicited, and he has remained dependent on family supervision for daily functioning despite preserved ability in basic self-care.

The patient’s medical history was notable for recurrent microcytic hypochromic anemia consistent with iron deficiency and persistently elevated serum IgE, alongside gastrointestinal disturbances. Additional investigations revealed hypercalcemia with inappropriately elevated parathyroid hormone level of 96 ng/L (reference range 15-65 ng/L) and subclinical hypothyroidism with thyroid nodules classified as Thyroid Imaging Reporting and Data System (TI-RADS) 2. At a later stage, he developed acute somatic complaints of nausea, vomiting, diarrhea, and intermittent abdominal pain, which coincided with marked neuropsychiatric deterioration characterized by insomnia, diurnal and nocturnal incontinence, and behavioral dysregulation, necessitating inpatient admission. Although vesicoureteral reflux and other urinary tract anomalies have been reported in TBRS, no renal ultrasound was performed, as there were no localizing urinary symptoms or prior evidence of infection. Urinalysis and urine culture during hospitalization were unremarkable, and renal function tests were within normal limits. The urinary symptoms were therefore attributed to behavioral and neurodevelopmental regression rather than an anatomical abnormality.

**Figure 1:**
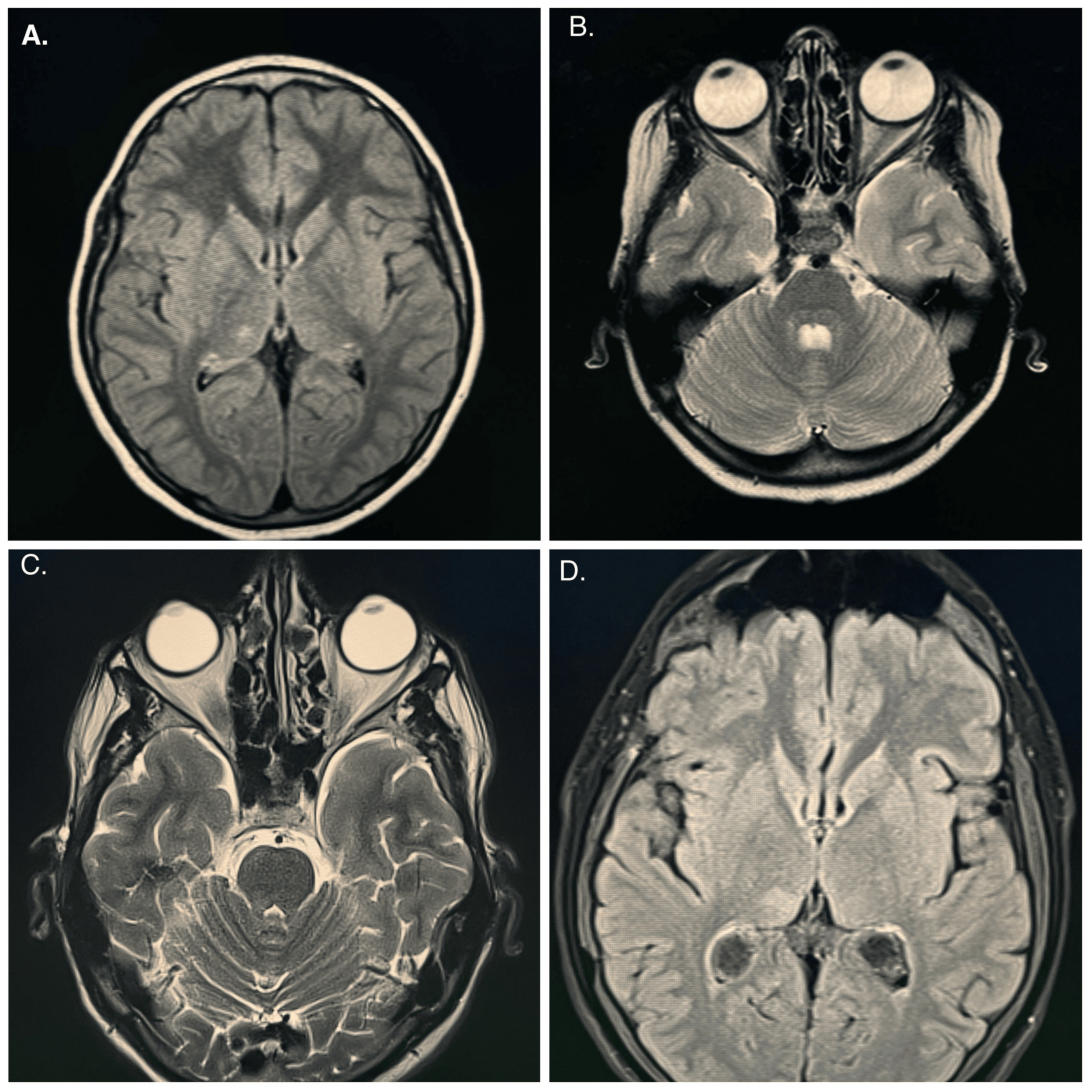
Axial fluid-attenuated inversion recovery (FLAIR) MRI brain images from 2012 (A-B) and 2025 (C-D) showing a stable, non-enhancing hyperintense lesion in the right thalamus, consistent with a long-standing benign structural finding.

Serial brain MRI studies between 2012 and 2025 revealed a stable, non-enhancing T2-weighted fluid-attenuated inversion recovery (T2/FLAIR) hyperintense lesion localized to the right thalamus with subtle extension into the right cerebral peduncle and facial colliculus (Figure 1). There was no diffusion restriction, blooming artifact, mass effect, or hydrocephalus, and MR spectroscopy demonstrated a normal metabolic profile. No additional intracranial lesions were identified, and the abnormality has remained radiologically stable for more than a decade, supporting a benign, non-progressive structural anomaly rather than a neoplastic process.

**Figure 2:**
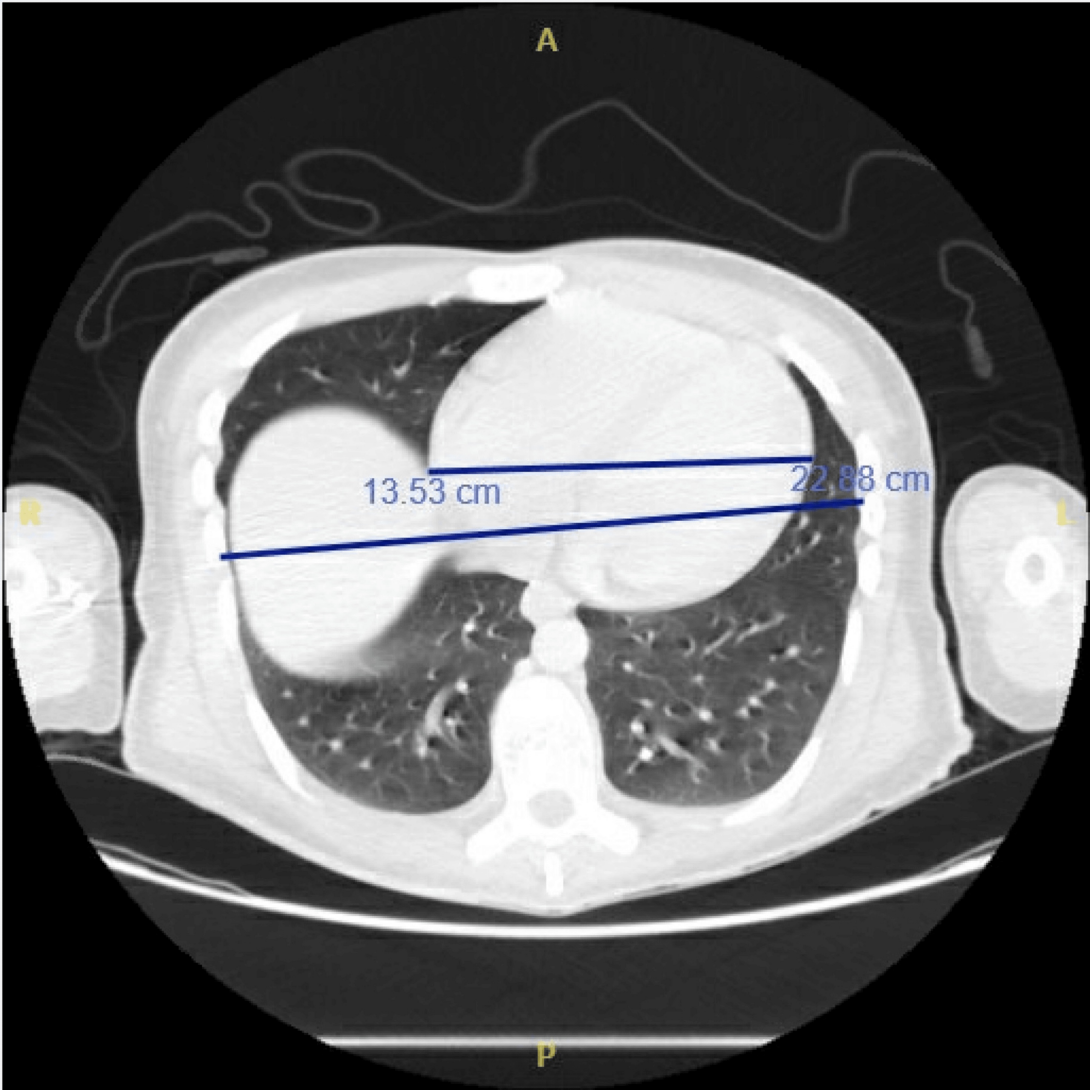
Axial contrast-enhanced chest CT showing mild cardiomegaly (cardiothoracic ratio ≈ 0.59) and borderline aortic root dilatation (3.7 cm).

Chest CT with contrast demonstrated mild cardiomegaly, with a transverse cardiac diameter of 13.53 cm and a thoracic width of 22.88 cm, yielding a cardiothoracic ratio of approximately 0.59, consistent with borderline enlargement (Figure 2). The study also showed aortic root dilatation measuring 3.7 cm, while the pulmonary trunk and cardiac chambers were within normal limits. There was no evidence of pericardial effusion, valvular calcification, or acute pulmonary embolism. The findings were interpreted as mild cardiomegaly with borderline aortic root dilatation, and periodic echocardiographic follow-up was advised.

Genetic evaluation was pursued due to the patient’s overgrowth phenotype, global developmental delay, and dysmorphic features. Whole-exome sequencing revealed a heterozygous missense variant in the DNMT3A gene (c.923G>T, p.Gly308Val), confirmed by Sanger sequencing. This variant was initially classified as a variant of uncertain significance, and parental segregation testing was recommended to determine whether it had occurred de novo. Although the de novo status was not genetically confirmed, the overall clinical presentation was consistent with TBRS. The phenotype included tall stature, overgrowth, macrocephaly, coarse facial features with a broad forehead and thickened eyebrows, together with mild intellectual disability, ASD, ADHD, and behavioral disturbances. The constellation of these neurodevelopmental and physical findings strongly supports the diagnosis of TBRS associated with the identified DNMT3A variant.

During hospitalization, the patient was managed through a multidisciplinary team approach involving psychiatry, gastroenterology, endocrinology, neurology, cardiology, and genetics, ensuring coordinated care for his complex neuropsychiatric and systemic manifestations.

## Discussion

This case highlights the expanding neuropsychiatric and systemic features of TBRS. In addition to the known OGID, our patient also showed signs of co-occurring ASD, ADHD, developmental regression in early adulthood, and psychotic-like behaviors. These findings emphasize the complexity of long-term outcomes associated with this syndrome.

Intellectual disability in TBRS is almost universal, though its severity varies. In the largest clinical cohort, most individuals showed mild to moderate impairment, while a smaller proportion fell into the severe range [3]. In their case series providing the first systematic neuropsychological assessment of TBRS, Lane et al. reported that affected individuals often show an uneven cognitive profile, with relative strengths in verbal abilities when compared to non-verbal reasoning and spatial skills [7]. Our patient’s marked discrepancy between verbal and performance IQ mirrors this pattern and highlights the importance of recognizing cognitive heterogeneity in TBRS. Such variability carries direct educational implications: relative verbal strengths can be leveraged in individualized learning programs, while additional support is needed for visuospatial and problem-solving domains. Consistent with the recommendations outlined by Ostrowski and Tatton-Brown [4], systematic neurocognitive assessment should be incorporated into routine care to guide educational planning and monitor developmental changes over time.

In the largest clinical cohort, behavioral and psychiatric issues were reported in 51% (28/55) of individuals, with ASD present in 36% (20/55) as the most frequent co-occurring diagnosis [3]. In their case series, Lane et al. complemented this by administering standardized tools (e.g., ADOS-2), showing autistic traits are prevalent alongside intellectual disability and adaptive skill limitations, with a characteristic uneven cognitive profile (verbal strengths > non-verbal/spatial) [7]. Our patient was formally diagnosed with ASD and demonstrated relative verbal strengths with weaker performance IQ, aligning closely with the cognitive profile described in TBRS. Pharmacological interventions are generally reserved for severe behavioral complications; consistent with broader ASD treatment literature, risperidone and aripiprazole have demonstrated efficacy for irritability, self-injuries, and aggressive behaviors in children and adolescents with ASD [8,9]. Thus, our patient’s clinical course aligns with reported ASD features in TBRS, and his management highlights the importance of syndrome-informed assessment and evidence-based interventions that address both core developmental needs and associated behavioral challenges.

Although less frequently reported than ASD, ADHD has been described in TBRS. In the large clinical cohort, behavioral difficulties were common, but specific ADHD diagnoses were not the predominant feature [3]. In their case series, Lane et al. highlighted attentional control and executive-function weaknesses as part of the broader neurocognitive profile, which may explain the emergence of ADHD symptoms in a subset of affected individuals [7]. A concrete example was provided by Martin et al. in their case report describing a patient with TBRS and ADHD who responded well to methylphenidate [2]. In our case, childhood hyperactivity and distractibility responded well to stimulant therapy, echoing the findings of Martin et al., who reported a TBRS patient with ADHD successfully treated with methylphenidate [2]. This approach is consistent with recommendations that management remain symptom-focused, applying conventional strategies where indicated [4]. The convergence of our experience with prior reports strengthens the view that ADHD in TBRS should not be overlooked, and when identified, should be managed proactively using evidence-based methods adapted to the individual’s developmental and cognitive context.

A distinctive feature in this patient was the emergence of regression and psychotic-like symptoms in early adulthood. Neurodevelopmental regression and psychotic disorders, including schizophrenia, have been documented in DNMT3A-related TBRS, prompting recommendations for lifelong psychiatric follow-up [1]. Recent neuroimaging work by Jiménez de la Peña et al. demonstrated structural brain alterations, such as increased cortical thickness and corpus callosum changes, that may underlie neuropsychiatric manifestations in TBRS [5]. Lane et al., in their case series, did not observe these outcomes but acknowledged prior reports [7]. Our patient’s loss of language, decline in self-care, incongruent affect, and persistent self-talk parallel the spectrum described by Tenorio et al. in their study and support this need for surveillance [1]. In ASD, regression and late-emerging behavioral changes may resemble psychosis, yet differentiating stereotyped self-dialogue or inner speech from true hallucinations is clinically challenging [10]. Psychotic experiences are also more common in autistic individuals than in the general population [11,12], and may present with functional deterioration, as in our case. Thus, his trajectory reflects both the recognized TBRS neuropsychiatric spectrum and the broader vulnerability of ASD populations to regression and psychotic-like symptoms, underscoring the importance of repeated, collateral-rich assessments and close longitudinal monitoring [1,7,10-12].

The genotype-phenotype relationship in TBRS remains an area of active study. Missense variants of DNMT3A are more common and frequently cluster in functional domains than nonsense, frameshift, or splice variants [3,11]. Our case introduces a previously unreported missense variant, p.Gly308Val, located in the PWWP domain, which is consistent with prior reports that pathogenic missense changes frequently involve functional regions of DNMT3A [3,5]. The patient’s phenotype-overgrowth, macrocephaly, intellectual disability, and neuropsychiatric features mirror the established spectrum, supporting the pathogenic relevance of this variant. Similar reports of novel DNMT3A variants from Latin America and East Asia highlight that TBRS is not restricted to specific populations and that each new case contributes to refining genotype-phenotype correlations [2,5,13]. 

In this case, normocytic anemia occurred alongside marked weight loss and biopsy-proven gastrointestinal disease (*Helicobacter pylori* gastritis and active proctitis), suggesting a multifactorial, secondary etiology (nutritional/inflammatory/occult blood loss) rather than a primary feature of TBRS. Urinary incontinence was also noted in our patient without evidence of infection or structural abnormality on evaluation. Although vesicoureteral reflux, cryptorchidism, and other genitourinary anomalies have been reported sporadically in TBRS cohorts [1,3], these are considered non-core findings. The absence of anatomic pathology in our case, together with the timing of symptom onset during behavioral regression, suggests a functional or neurodevelopmental origin rather than a structural urinary tract defect. Large TBRS series and syntheses do not identify anemia as a consistent phenotypic element, although the role of DNMT3A in hematopoiesis and the reported leukemia risk justify routine hematologic surveillance [3,4]. Our patient also exhibited eczema with elevated IgE, features not described in the largest TBRS cohort [3] nor included by Ostrowski and Tatton-Brown [4]. Although these manifestations are not considered part of the core phenotype, their occurrence raises the possibility of underrecognized atopic or immune dysregulation in TBRS. DNMT3A plays a central role in epigenetic regulation of hematopoiesis and immune cell differentiation [3,5], and it is conceivable that variants in functional domains, such as the novel missense change in our case, may contribute to immune or dermatologic vulnerability. Further accumulation of cases will be needed to clarify whether eczema and elevated IgE represent coincidental comorbidities or reflect a broader, incompletely defined spectrum of DNMT3A-related disease.

Although not part of the core phenotype, cardiovascular abnormalities have been reported in several TBRS cohorts and isolated case descriptions. In the largest clinical study of 55 individuals, Tatton-Brown et al. identified a subset of patients with aortic root dilatation and valvular anomalies, including mitral and tricuspid valve prolapse [3]. Tenorio et al. similarly documented mild aortic dilatation and valve thickening in individual cases, recommending routine cardiac evaluation [1]. Our patient’s CT findings of mild cardiomegaly and aortic root dilatation (3.7 cm) parallel these observations and suggest that DNMT3A-related dysregulation may extend to connective-tissue or vascular structures. While the precise mechanism remains unclear, DNMT3A plays a role in vascular smooth-muscle differentiation and epigenetic control of extracellular-matrix remodeling, which could predispose to aortic wall elasticity changes. Given the potential for progressive enlargement similar to that observed in other overgrowth syndromes such as Sotos and Weaver, ongoing echocardiographic monitoring is warranted. Recognizing these cardiovascular features expands the systemic spectrum of TBRS and underscores the importance of multidisciplinary surveillance incorporating cardiology follow-up in long-term management.

Management requires a broad multidisciplinary approach. Care is multidisciplinary and symptom-focused, with periodic review of growth, development, and behavior, and conventional treatments for co-occurring neuropsychiatric conditions [4]. Given sporadic reports of TBRS complicated by hematologic malignancies, clinical vigilance is advisable, though the precise lifetime risk is not established [4].

From a regional standpoint, this appears to be the first reported TBRS case from Saudi Arabia that we are aware of. Prior publications document TBRS across Europe, Latin America, and Asia (including Japan and Korea) [2,3,5,13,14]. Documenting this case may raise regional awareness and contribute to international datasets.

## Conclusions

This case expands the clinical, neuropsychiatric, and systemic spectrum of TBRS by highlighting its association with intellectual disability, ASD, and additional findings, including stable right thalamic signal abnormality and aortic root dilatation. It underscores the importance of multidisciplinary assessment and longitudinal neuropsychiatric follow-up in TBRS. Continued reporting of similar cases will be essential to refine genotype-phenotype correlations and guide comprehensive long-term management.
